# BEV 2C protein inhibits the NF-κB signalling pathway to promote viral replication by targeting IKBKB and p65

**DOI:** 10.1186/s13567-025-01453-8

**Published:** 2025-02-16

**Authors:** Xuyuan Cui, Yidi Guo, Fan Zhang, Xiaoran Chang, Junying Hu, Qun Zhang, Xuebo Zheng, NaiTian Yan, Xinping Wang

**Affiliations:** State Key Laboratory for Diagnosis and Treatment of Severe Zoonotic Infectious Diseases, Key Laboratory for Zoonosis Research of the Ministry of Education, Institute of Zoonosis, and College of Veterinary Medicine, Jilin University, Changchun, China

**Keywords:** BEV, HY12 enterovirus, 2C protein, NF-κB, p65, IKBKB, enterovirus replication

## Abstract

Bovine enterovirus, a member of the *Enterovirus* genus in the *Picornaviridae* family, causes severe digestive and respiratory illnesses in cattle. These illnesses threaten the healthy development of the cattle industry. Innate immunity plays a critical role in resisting viral infections, but viruses also use various strategies to evade or counteract the host’s immune system. The mechanisms by which bovine enteroviruses evade the host immune response and promote their replication remain unclear. This study used the HY12 strain of enterovirus as a model to investigate its interaction with both bovine enterovirus and its host. Our findings indicate that bovine enterovirus promotes the replication of HY12 by disrupting the NF-κB pathway. Here, one strategy was to down-regulate the IΚBΚB expression to inhibit the activation of NF-κB. Another approach was to directly interact with p65 to reduce the dimerisation of p65/p50 and inhibit the phosphorylation and nuclear translocation of p65. Our study’s results show that 2C’s N-terminal 1-121 aa is essential for 2C-mediated inhibition of the NF-κB signalling pathway, and four amino acids (position 118-121 aa) are the interaction site of 2C with p65. This report is the first on BEV 2C protein promoting virus replication through new strategies, which provides novel insights into the understanding of enterovirus pathobiology and the development of drugs against BEV.

## Introduction

Bovine enterovirus (BEV) is a single-stranded, positive-sense RNA virus belonging to the genus *Enterovirus* within the family of *Picornaviridae* [1]. BEV infection causes severe digestive and respiratory diseases in cattle, resulting in a high mortality rate that incurs significant economic losses for the global cattle industry [2]. The genome of BEV is approximately 7400 nucleotides long. It contains an open reading frame encoding a multiprotein precursor, which is subsequently cleaved into four structural proteins (VP1, VP2, VP3, and VP4) and seven non-structural proteins (2A, 2B, 2C, 3A, 3B, 3C, and 3D) [3]. Structural proteins are predominantly involved in forming virion capsids and are responsible for eliciting immune responses, while non-structural proteins play important roles in viral replication [4, 5].

Out of the non-structural proteins, 2C is one of the most conserved and complex proteins, typically comprising 330 amino acid residues [6]. It contains an N-terminal membrane-bound domain, a central ATPase domain, a cysteine-rich domain, and a C-terminal helix domain [7]. This protein plays multiple biological functions in the viral life cycle, including viral uncoating [8], host cell membrane rearrangement [9, 10], RNA binding [11], RNA replication [12], capsidisation, and morphogenesis [13]. Studies have shown that the 2C proteins of Enterovirus A71 (EV-A71) and Coxsackievirus A16 (CV-A16) viruses possess ATP-dependent RNA helicase activity and ATP-independent chaperone activity, which are crucial for viral RNA replication [14]. Additionally, the 2C protein of EV-A71 has been found to interact with a host protein called Reticulon3, which affects the early stages of the virus’s replication within the host [15]. Furthermore, the 2C protein acts as a nucleoside-triphosphatase (NTPase), guiding replication complexes to reach the cell membrane to promote viral replication [16]. Despite the significant advances in understanding the structure and function of 2C, the relationship between 2C and host proteins and its connection to the host’s innate immune response remains unclear.

The innate immune system plays a key role in fighting viral infections. When the host recognises the pathogen-associated molecular patterns (PAMPs) through pattern recognition receptors (PRRs), the innate immune reaction for an antiviral response is activated through specific signalling pathways [17]. Notably, NF-κB is the critical innate immune pathway involved in the antiviral response. Furthermore, the activation of NF-κB can induce the expression of IFN-β, MHC class I, and several inflammatory cytokines [18]. The IκB kinase (IKK) complex is essential for activating NF-κB signalling in response to various stimuli, such as those derived from RNA or DNA [19, 20]. IKK complex consists of three subunits: IKKα (CHUK), IKKβ (IΚBΚB), and IKKγ. IKKα and IKKβ are the catalytic subunits, while IKKγ is the regulatory subunit [20].

Activation of the IKK complex leads to the phosphorylation of IκBα and its degradation through the ubiquitin–proteasome system. Subsequently, this process releases NF-κB transcription factors and initiates the nuclear translocation [21], which triggers the expression of multiple genes involved in eliciting innate or adaptive immune and inflammatory responses and limits viral replication [22–24]. Simultaneously, viruses employ certain strategies to evade the host’s immune response and sustain their replication. One strategy is balancing NF-κB activation in the process of confrontation with the host, as seen in the hepatitis C virus [25], paramyxovirus [26], influenza virus [27], African swine fever virus [28], poxvirus [29], and others.

Typically, these viruses encode proteins to disrupt or modulate immune responses by targeting specific aspects of the NF-κB signalling pathway. A similar mechanism has also been reported in some viruses of the *Picornaviridae* family. For example, the poliovirus inhibits TNF-α-mediated NF-κB activation by eliminating TNFR on the cell surface through its 3A protein [30]; Coxsackievirus B3 [31] and foot-and-mouth disease virus [32] inhibit NF-κB activation via viral protease proteins; and the 2C protein of EV-A71 reduces the phosphorylation of IΚBΚB and inhibits the activation of NF-κB by interacting with IΚBΚB [33].

Previously, we demonstrated that infection with the BEV HY12 strain causes a cytokine storm with increased levels of pro-inflammatory and inflammatory cytokines, including TNF-α [34, 35], which is one of the stimulators of the NF-κB activation pathway. We also found the dynamic expression patterns of cytokines such as IFNs and TNF-α negatively correlated with the virus replication or titre [36]. Consequently, we hypothesise that BEV may evolve strategies to interfere with TNF-α-induced NF-κB activation to evade the host’s immune response.

For this study, we employed multiple molecular biology and immunology approaches to explore the potential viral proteins that interact with host factors to facilitate viral replication and the underlying mechanism. We discovered that BEV 2C uses a new strategy to promote virus replication: either directly interacting with p65 to block the dimerisation of p65/p50 or inhibiting the activation of NF-κB signalling elicited by viral infection by suppressing the inhibitor of nuclear factor kappa B kinase subunit beta (IKBKB) expression. This strategy will help elucidate the pathogenesis of enteroviruses and provide new approaches for preventing and treating enterovirus infections.

## Materials and methods

### Cell cultures and virus propagation

Madin-Darby bovine kidney (MDBK) and Vero cells were cultured in Dulbecco’s modified Eagle’s medium (DMEM) (Invitrogen, Carlsbad, CA, USA), supplemented with 5% foetal bovine serum (HyClone, Beijing, China), 2 µg/mL gentamicin (Beyotime Biotechnology, Shanghai, China), and 2 mM l-glutamine (Beyotime Biotechnology). The 293T cells were cultured in DMEM (Invitrogen), supplemented with 10% foetal bovine serum (HyClone, Beijing, China), 2 µg/mL gentamycin, and 2 mM l-glutamine.

Enterovirus HY12 strains (GenBank accession no. KF748290.1) were isolated and kept in our laboratory [2]. HY12 virus propagation and titration were performed using Vero cells. Virus titration was conducted using Vero cells in 96-well plates and expressed as the 50% tissue culture infectious dose (TCID_50_) per unit volume, as described previously [37].

### Plasmid constructs and transfection

Plasmids expressing HY12-encoded proteins were constructed by cloning the corresponding fragments amplified from the EV strain HY12 (GenBank accession number: KF748290.1) by reverse transcription polymerase chain reaction (RT-PCR) into the PCI-neo vector (Flag-tagged encoded protein). The primers used in plasmid construction are shown in Table 1.

**Table 1:** **Primers used for plasmid construction**

Gene name	Forward primer sequence (5ʹ–3ʹ)	Reverse primer sequence (5ʹ–3ʹ)	
HY12-2C HY12-VP1 HY12-VP2 HY12-VP3 HY12-VP4 HY12-2A HY12-2B HY12-3AB HY12-3C HY12-3D 2C (aa 1–125) 2C (aa 126–253)	CCG**GAATTC**TCAGACAATTGGAT CCG**GAATTC**AATGACCCAGGGAA CCG**GAATTC**TCTCCGTCAGCAGAA CCG**GAATTC**GGCCTTCCAACAAA CCG**GAATTC**ATGGGCGGTCAGTT CCG**GAATTC**GGGCCTTTTGGACA CCG**GAATTC**GGCATTACTGATTAT CCG**GAATTC**GGCCCCGTAACCTA CC**CTCGAG**GGACCACTCTTTGA CCG**GAATTC**GGGCAAATAGAATAC GC**GTCGAC**TCAGACAATTGGATAA GC**GTCGAC**CTCATACATGGCTCTCC	ATTT**GCGGCCGC**TTATTGAAAAAGG ATTT**GCGGCCGC**GTACGAGGTGAG ATTT**GCGGCCGC**TTGGTTTGATGCAA ATTT**GCGGCCGC**CTGTAGTGCGGCTG ATTT**GCGGCCGC**TTTCAGCGGAACG ATTT**GCGGCCGC**TTGCTCCATAGCGT ATTT**GCGGCCGC**TTGCCTCTCTGCCA ATTT**GCGGCCGC**TTGAGTTTGAACCT ATTT**GCGGCCGC**TTGTTCAACAGTAA ATTT**GCGGCCGC**TTAGAAAGAATCT CG**GGATCC**TTATGCACATACGGGTTC CG**GGATCC**TTATGTCTTTCTGTACT	
2C (aa 254–329) 2C (aa 1–121) 2C (aa 1–117) 2C (aa 1–113) p65 p65 (aa 1–290) p65 (aa 291–551) p65 (aa 1–194) p65 (aa 195–290) IKBKB	GC**GTCGAC**TATAAGAAGAATGGAT GC**GTCGAC**TCAGACAATTGGATAA GC**GTCGAC**TCAGACAATTGGATAA GC**GTCGAC**TCAGACAATTGGATAA CGC**GGATCC**ATGGACGAACTGTTCC CGC**GGATCC**ATGGACGAACTGTTCC CG**GGATCC**ATGGATACAGACGATC CGC**GGATCC**ATGGACGAACTGTTCC CGC**GGATCC**ATAAGATCTGCCGAG CC**AAGCTT**ATGAGAAGGCTGACCC	CG**GGATCC**TTATTGAAAAAGAGCCTC CG**GGATCC**TTATTCAATGCGATTC CG**GGATCC**TTACTTGGTCTTGAACTG CG**GGATCC**TTACTGCATGGCACCGAG TGC**TCTAGA**GGAGCTGATCTGACT TGC**TCTAGA**TGGCAGGTACTGGAATT TGC**TCTAGA**GCTGATCTGACTCAGC TGC**TCTAGA**GAGCTCGGCAGTGTT TGC**TCTAGA**TGGCAGGTACTGGAATT GC**TCTAGA**TGAGGCCTGCTCCAGG	

The restriction enzyme cutting sites are highlighted in bold.

Expression plasmids containing the full-length and truncated p65 gene were generated by inserting the PCR-amplified corresponding fragments from 293T cells into the *Bam*H I and *Xba *I sites of the pcDNA3.1 vector (His-tagged p65 or His-tagged truncated p65). The full-length IKBKB gene (GenBank: NM_001190720) was amplified from the 293T cells and cloned into the *Bam*H I and *Eco*R I sites of the pcDNA3.1 vector (Invitrogen) to generate His-IKBKB protein (His-tagged IKBKB).

According to the standard procedure, plasmids containing 2C and 2C truncated fragments were constructed on the PCI-neo vector. These plasmids were Flag-tagged 2C. Luciferase reporter plasmid pNF-κB-Luc containing κB binding motifs, luciferase reporter gene (Luc), and control plasmid pRL-SV40 were purchased from Beyotime Biotechnology. The corresponding plasmids were transfected using polyethyleneimine (PEI) (Polysciences, Warrington, PA, USA) according to the manufacturer’s instructions for transfection experiments.

### Reporter gene assays

Firefly luciferase and Renilla luciferase activities were detected using the Dual-Luciferase Reporter Gene Assay Kit (Beyotime Biotechnology). Cell viability assays were conducted following the manufacturer’s instructions using the Calcein AM Cell Viability Assay Kit (CCK-F) (Beyotime Biotechnology). All reporter gene assays were repeated at least three times. Data are presented as the mean ± SD values.

### Confocal microscopy

The 293T cells were plated on 24-well plates with coverslips. After being fixed with methanol at −20 °C for 20 min, the cells were blocked with 5% skim milk for 30 min. They were then incubated with the indicated primary antibodies for 1 h and probed with Alexa Fluor 594-labelled goat anti-mouse IgG and/or Dylight 488-labelled goat anti-rabbit IgG antibodies.

Cell nuclei were stained with a 1 μg/mL 4′,6-diamidino-2-phenylindole (DAPI) (Roche) methanol solution. After mounting with an autofluorescence quenching mountant, the slides were observed by a confocal microscope (Fluoview FV3000; Olympus, Tokyo, Japan).

### Immunoprecipitation and immunoblot analysis

Cells were lysed using radioimmunoprecipitation assay (RIPA) lysis buffer (Epizyme, Shanghai, China) containing a mixture of protease inhibitors and phosphatase inhibitors. The cell lysates were sonicated before centrifugation at 10 000 rpm for 20 min at 4 °C. The lysates were subjected to overnight immunoprecipitation using either mouse anti-Flag Magnetic Beads or mouse anti-His Magnetic Beads. The protein concentration of each sample was determined by a Bicinchoninic Acid Assay (BCA) protein assay kit (Epizyme, Shanghai, China). An equal amount of total protein was loaded, separated by SDS-PAGE.

After the proteins were transferred onto the polyvinylidene fluoride membranes (Millipore, MA, USA), the membranes were blocked in 5% skim milk and incubated with the primary antibodies before being probed with the corresponding secondary antibodies conjugated with horseradish peroxidase (HRP). Subsequently, after washing, the electrochemiluminescence (ECL) reagent (Yeasen, Shanghai, China) was used for the chemiluminescent detection of target proteins following the manufacturer’s instructions. The protein band was visualised using a Luminescent Image Analyser (Tanon 4600SF, Shanghai, China). Equal loading of the proteins was normalised by β-tubulin.

### Real-time reverse transcription polymerase chain reaction (qPCR)

The cDNAs were synthesised using ABScript III RT Master Mi × for qPCR with gDNA Remover (ABclonal Technology, Wuhan, China). They were then used for PCR amplification using a 2 × Universal SYBR Green Fast qPCR Mix (ABclonal Technology, Wuhan, China). The relative mRNA abundance was normalised to glyceraldehyde-3-phosphate dehydrogenase (GAPDH) expression and evaluated by the 2^−ΔΔCT^ method [38]. Real-time PCR primers were designed using Primer-BLAST, and their sequences are listed in Table 2.

**Table 2:** **Primers used for qPCR**

Gene name	Forward primer sequence (5ʹ–3ʹ)	Reverse primer sequence (5ʹ–3ʹ)
h-GAPDH HY12-VP1 h-IL-8 h-IL-1β h-TNF-α h-IKBKB	GACAAGCTTCCCGTTCTCAG CCACTGATGCAACACCCGCTCTA GGTGCAGTTTTGCCAAGGAG ATGGCTTATTACAGTGGCAATGAGG TGGCGTGGAGCTGAGAGATAAC TCCAGATCATGAGAAGGCTGAC	GAGTCAACGGATTTGGTGGT CGCTTGTTTCATGTATGCCGTGTG TTCCTTGGGGTCCAGACAGA AGTGGTGGTCGGAGATTCGTAG GCTGATGGTGTGGGTGAGGAG CCAAGTTCTGCATCCCCTCA

h represents human.

### Statistical analysis

Statistical analyses were performed using GraphPad Prism 8.0 software. An unpaired Student *t*-test was used to assess the statistical significance of comparing two means. A one-way analysis of variance** (**ANOVA) test was used for dose-dependent experiments or multiple comparisons, followed by a post hoc test (Dunnett or Tukey test). Quantitative data in histograms are shown as means ± SD. Statistical relevance was evaluated using the following *p*-values: *p < 0.05, **p < 0.01, ***p < 0.001.

## Results

### BEV inhibits TNF-α-triggered activation of the NF-κB

TNF-α plays a central role in host defence against viral infection. Previously, we showed how BEV triggers elevated levels of TNF-α in the early stages of infection and expression of TNF-α is negatively correlated with the virus replication or virus titre [39]. Therefore, we hypothesised that BEV may also develop strategies to interfere with TNF-α-induced NF-κB activation during the later stages of infection to evade host immune surveillance.

**Figure 1:**
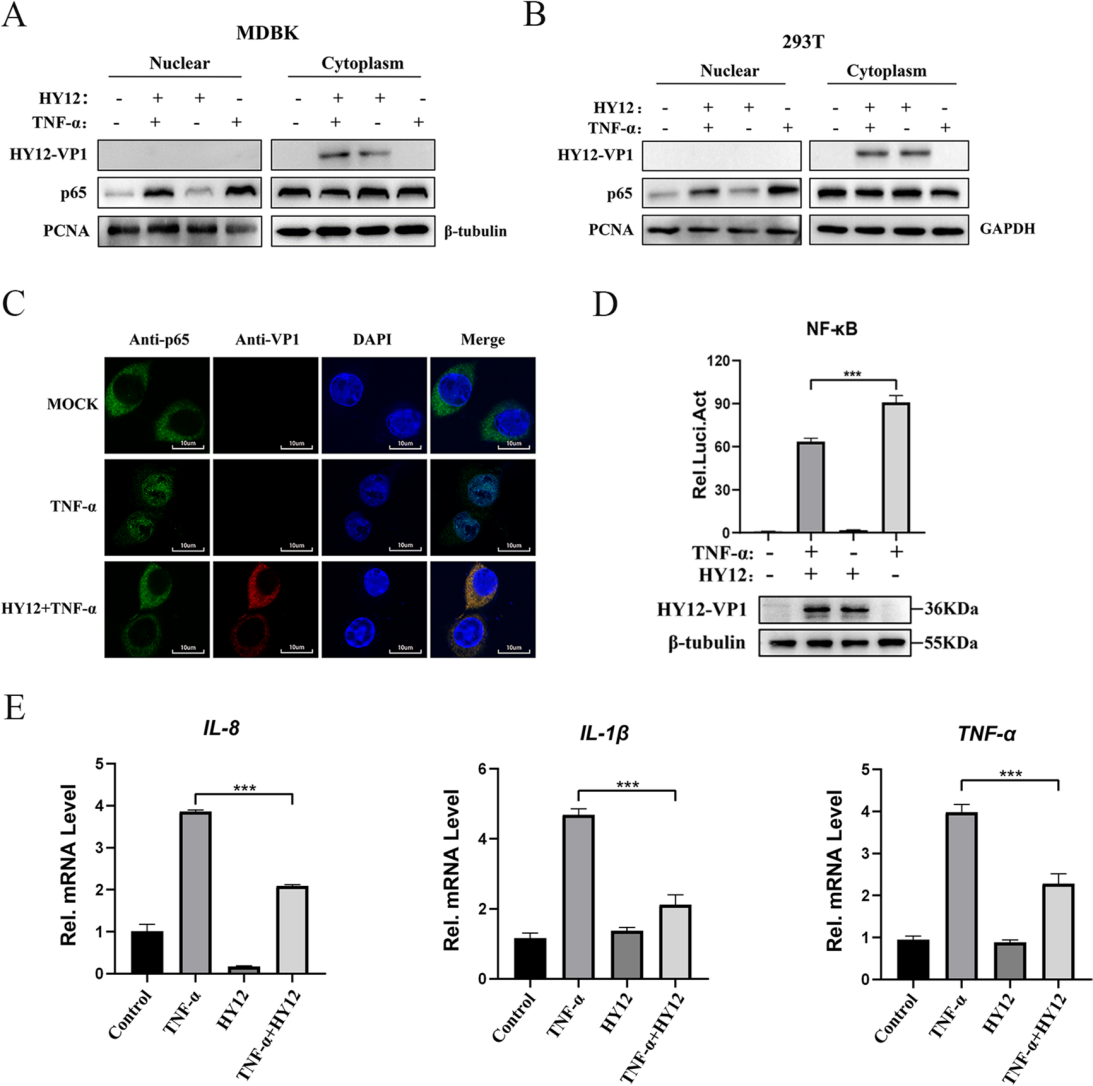
**BEV inhibits TNF-α-triggered activation of the NF-κB**. **A**–**C** HY12 inhibits TNF-α-triggered nuclear translocation of p65. MDBK cells (**A**) and 293T (**B**) cells were mock infected or infected with HY12 viruses at a MOI of 0.5. Ten hours post-infection, mock- and HY12-infected cells were treated with or without TNF-α for 20 min at a concentration of 20 ng/mL. Cell lysates were then separated into cytoplasmic and nuclear fractions. Both cytoplasmic and nuclear proteins were analysed by western blot with anti-p65 Ab to reveal the localisation of NF-κB. Nucleus-specific anti-PCNA Ab and cytoplasmic-specific anti-tubulin or GAPDH Ab were used as controls. **C** Localisation analysis of p65 was performed by confocal microscopy using FV3000 software. 293T cells were treated in the same way as described in **A**. A confocal microscope was used to localise p65 (green) and HY12-VP1 (red). Cell nuclei were stained with 4,6-diamidino-2-phenylindole (DAPI) (blue). Scale bar, 10 μm. **D**–**E** HY12 inhibits TNF-α-induced NF-κB activation. **D** 293T cells were co-transfected with NF-κB-Luc reporter plasmid (0.4 μg) and pRL-SV40 plasmid (0.08 μg). Twenty-four hours post-transfection, the cells infected by BEV HY12 (MOI of 0.5) for 10 h or mock-infected were treated with TNF-α (20 ng/mL) for 6 h and harvested for luciferase reporter gene assays. (E) Expression levels of mRNA for IL-8, IL-1β, and TNF-α were measured by qPCR with GAPDH as an internal reference gene. Results are representative of three independent experiments. Data are presented as mean ± SD values. *P*-values < 0.05 (*), < 0.01 (**), and < 0.001 (***) were considered statistically significant and highly significant, respectively.

To test this hypothesis, we determined the nuclear translocalisation activity of p65 using immunoblotting and a confocal microscopy. Western blotting results indicated that p65 was significantly reduced in the nucleus of MDBK cells infected with the HY12 virus compared to mock-infected cells after TNF-α stimulation conditions (Figure 1A). We observed similar results in 293T cells (see Figure 1B). When exposed to the same TNF-α stimulation conditions, confocal microscope analysis revealed that p65 protein expression predominantly accumulated in the cytoplasm of EV-infected cells. In contrast, it was primarily located in the nucleus of mock-infected cells (Figure 1C). These results suggest that HY12 infection inhibited TNF-α-mediated p65 nuclear translocation.

To determine BEV’s capacity to inhibit TNF-α-triggered activation of NF-κB signalling, 293T cells were transiently transfected with the pNF-κB-Luc reporter plasmid along with pRL-SV40 for 24 h before they were infected with HY12 for 10 h. The cells were treated with TNF-α for 20 min, after which they were collected to assay the luciferase activity. The level of luciferase activity of the BEV-infected cells was significantly lower than that of the mock-infected cells (Figure 1D), suggesting BEV virus infection results in the inhibition of the NF-κB signalling pathway.

To further investigate whether BEV is involved in regulating endogenous NF-κB signalling, we measured the transcriptional levels of inflammatory cytokines IL-8, IL-β, and TNF-α. Quantitative RT-PCR (RT-qPCR) demonstrated that HY12 significantly inhibited the TNF-α-induced mRNA expression levels of IL-8, IL-β, and TNF-α genes (Figure 1E).

The above results demonstrated that BEV significantly inhibits the TNF-α-triggered NF-κB signalling pathway.

### BEV 2C protein inhibits NF-κB activation to promote viral replication

To identify which viral proteins have the potential to regulate inflammatory responses, we used luciferase reporter assays to screen the 11 HY12-encoded structural or non-structural proteins for their ability to regulate the TNF-α-triggered NF-κB signalling.

**Figure 2:**
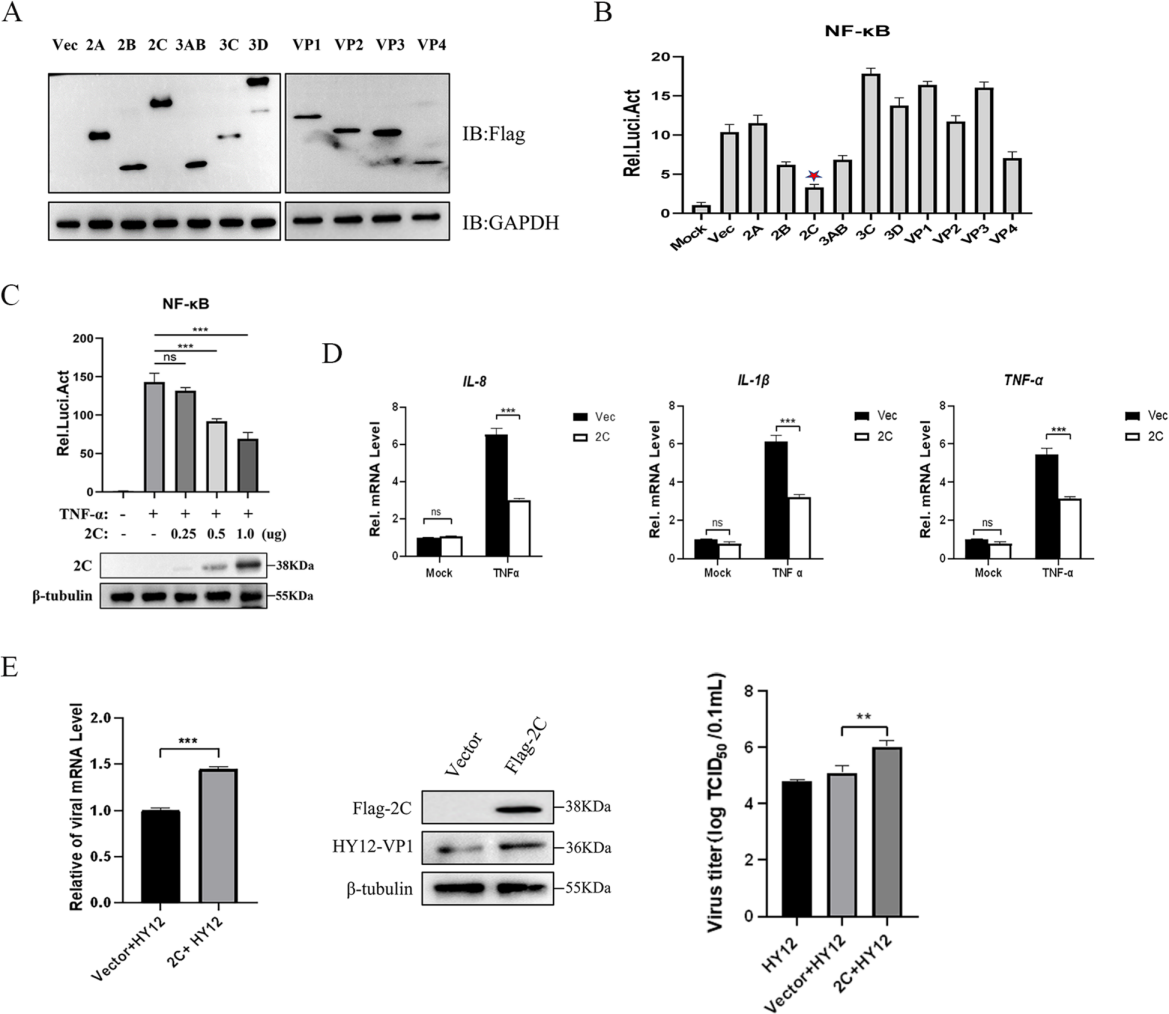
**BEV 2C protein inhibits the TNF-α-triggered NF-κB activation to promote viral replication.** **A** Expressions of BEV-encoded proteins were shown using a western blot assay. **B** BEV 2C protein inhibits TNF-α-triggered activation of the NF-κB promoter. 293T cells were transfected with pNF-κB-Fluc (0.4 μg), pRL-TK (0.08 μg), and the plasmids expressing different BEV HY12 proteins (2A, 2B, 2C, 3AB, 3C, 3D, VP1, VP2, VP3, VP4) (1 μg). Twenty hours post-transfection, the cells were treated with TNF-α (20 ng/mL) for 6 h before they were collected for luciferase assays. 2C protein was marked as ★. **C** BEV 2C protein inhibits the TNF-α-triggered activation of the NF-κB promoter in a dose-dependent manner. 293T cells were transfected with 2C expressing plasmid at different concentrations (0, 0.25, 0.5 or 1.0 μg) along with pNF-κB-Fluc (0.4 μg) and pRL-TK (0.08 μg). Twenty-four hours post-transfection, the luciferase assay and immunoblotting analysis were performed after the cells were treated with TNF-α (20 ng/mL) for 6 h. **D** BEV 2C inhibits the TNF-α-triggered transcription of pro-inflammatory cytokines. 293T cells were transfected with either the PCI-neo (Vec) or the PCI-Flag-2C (1.0 μg) for 24 h. RT-qPCR was performed to determine the expression of TNF-α, IL-1β, and IL-8 after treating the transfected cells with TNF-α (20 ng/mL) for 6 h. **E** BEV 2C protein promotes viral replication. 293T cells were transfected with either the PCI-neo (Vec) or the PCI-Flag-2C (1.0 μg) for 24 h, followed by BEV HY12 infection for 24 h. The expression level of viral VP1 mRNA was detected by qPCR. Expressions of Flag-2C, HY12-VP1, and β-Tubulin protein were detected by western blotting. Viral titres in cell supernatants at 36 hpi were determined and expressed as TCID_50_/0.1 mL. Results are representative of three independent experiments.

After the expression of HY12-encoded proteins was validated by western blotting (Figure 2A), 2C showed the maximal inhibition effect on the TNF-α-triggered activation of the NF-κB promoter (Figure 2B). The inhibition of 2C on the NF-κB promoter occurred in a dose-dependent manner (Figure 2C). To investigate whether 2C regulates endogenous NF-κB signalling, we measured the transcription levels of several pro-inflammatory cytokines following treatment with TNF-α in 293T cells overexpressing 2C. Quantitative RT-PCR (RT-qPCR) analysis revealed that ectopic expression of 2C significantly inhibited the mRNA expression levels of IL-8, IL-1β, and TNF-α (Figure 2D). These results suggest that 2C inhibits the TNF-α-triggered NF-κB signalling pathway and is involved in regulating the expression of pro-inflammatory cytokines triggered by TNF-α.

Since BEV 2C antagonises the TNF-α mediated antiviral response, the effect of 2C on BEV replication was further investigated. Therefore, we measured the mRNA and protein expression levels for HY12 VP1. Our results showed that the overexpression of 2C enhanced the replication of HY12 compared to empty vector-transfected cells (Figure 2E). In addition, the titre of HY12 viruses increased in cells overexpressing 2C compared to the control group transfected with empty vector (Figure 2E).

These outcomes indicate that 2C plays a positive regulatory role in BEV replication.

### BEV 2C protein inhibits NF-κB activation by suppressing the expression of IΚBΚB

**Figure 3:**
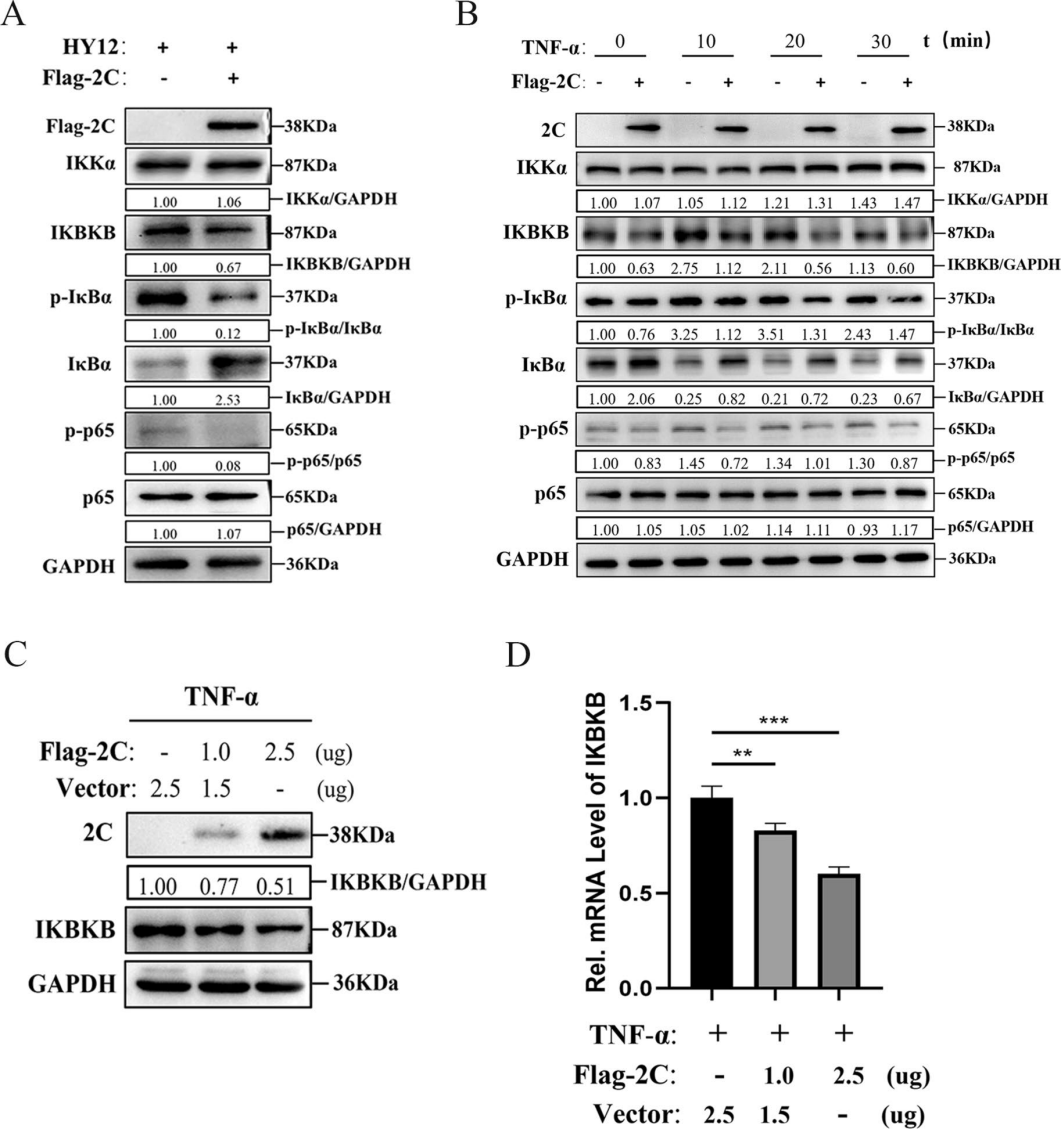
**BEV 2C protein inhibits NF-κB activation by reducing the expression of IΚBΚB**. **A** Overexpression of 2C protein inhibits the elevated expression of IΚBΚB by HY12 virus infection in 293T cells. 293T cells were transfected with PCI-neo (2.5 μg) or PCI-neo-2C (2.5 μg) for 24 h, followed by infection of HY12 viruses at a MOI of 0.5 for 12 h. The expressions of Flag-2C, HY12-VP1, IΚBΚB, CHUK, IκBα, p-IκBα, p-p65, p65 and GAPDH proteins were detected by western blot assay. **B** Overexpression of 2C protein inhibits TNF-α-mediated elevated expression of IΚBΚB in 293T cells. 293T cells transfected with PCI-neo or PCI-neo-2C for 48 h were treated with TNF-α (20 ng/mL) for 10, 20 or 30 min. Immunoblotting analysis was performed using the lysed cells. **C**, **D** 2C inhibits TNF-α-mediated elevated expression of IΚBΚB in a dose-dependent manner. 293T cells transfected with PCI-neo (Vector) (2.5, 1.5, 0 μg) and PCI-neo-2C (0, 1.0, 2.5 μg) for 48 h were treated with TNF-α (20 ng/mL) for 20 min. western blot analyses detected the expressions of Flag-2C, IΚBΚB and GAPDH protein. The mRNA expression level of IΚBΚB was measured via qPCR. GAPDH was used as an internal reference gene. Results are representative of three independent experiments. Data are presented as mean ± SD values. *P*-values < 0.05 (*), < 0.01 (**), and < 0.001 (***) were considered statistically significant and highly significant, respectively.

In response to the above findings that 2C inhibits the NF-κB activation and promotes BEV replication, we subsequently explored the underlying mechanisms of this process. To identify potential molecules targeted by the 2C protein, we examined the expression levels of key proteins (IΚBΚB, CHUK, IκBα, p-IκBα, p65, and p(phosphorylated)-p65) in the NF-κB signalling pathways by western blot assay. The expression levels of IΚBΚB, p-IΚBα, and p-p65 were significantly decreased in infected cells overexpressing 2C compared with the infected cells transfected with empty vector. In contrast, the expression levels of IΚBα were significantly increased (Figure 3A). These results demonstrated that overexpression of 2C indeed affects the expression of molecules in the NF-κB signalling pathway.

To further investigate if 2C has a similar effect on TNF-α-triggered NF-κB signalling, western blot analysis was performed on 293T cells treated with TNF-α at different times (10, 20, 30 min). Results indicated that 2C also suppressed the TNF-α-triggered NF-κB signalling, leading to the down-regulation of IΚBΚB expression (Figure 3B). Notably, the protein expression of IΚBΚB decreased with the increase of 2C expression (Figure 3C). Simultaneously, the mRNA levels for IΚBΚB were decreased in a dose-dependent manner by 2C overexpression (Figure 3D).

The above results suggest that BEV 2C inhibits NF-κB activation by down-regulating IΚBΚB expression.

### BEV 2C directly interacts with the p65

As a transcriptional factor, p65 is a key component of NF-κB transcriptional factor complexes involved in multiple cellular processes. Therefore, we hypothesised that 2C, which inhibits NF-κB activation, might interact with p65 to modulate the host immune response. Such an interaction could benefit virus survival.

**Figure 4:**
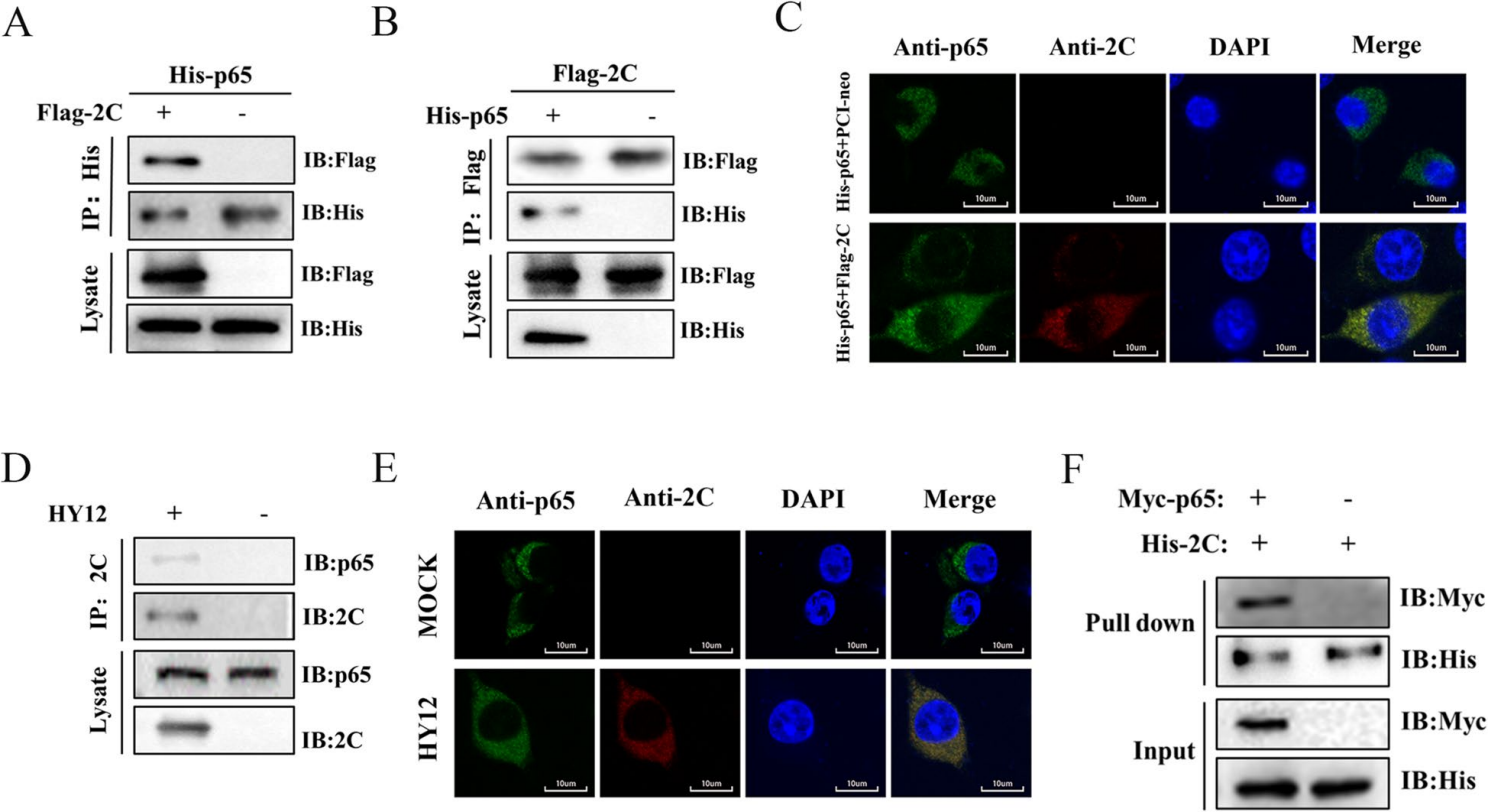
**BEV 2C interacts directly with p65.** **A** Interactions of 2C with p65 detected by Co-IP. 293T cells co-transfected with His-tagged p65 (6.0 μg) and Flag-tagged 2C (6.0 μg) or Flag-vector were immunoprecipitated using anti-His or anti-Flag antibody. Interactions between 2C and p65 were detected with immunoblotting. **B** 293T cells co-transfected with Flag-tagged 2C (6.0 μg) and His-tagged P65 (6.0 μg) or His-vector were immunoprecipitated using anti-Flag or anti-His antibody. Interactions between 2C and p65 were detected with immunoblotting. **C** Co-localisation of Flag-tagged 2C (0.5 μg) (red) and His-tagged p65 (0.5 μg) (green) was analysed in 293T cells co-transfected with Flag-2C and His-p65 plasmids for 48 h. 293 cells co-transfected of His-p65 with PCI-vector were used as a negative control. Cell nuclei were stained in DAPI (blue). Scale bar, 10 μm. **D** Cells infected with or without HY12 virus were immunoprecipitated with anti-2C and anti-p65 antibodies. The endogenous interaction between 2C and p65 was detected by western blotting. **E** Co-localisation of 2C (red) with p65 (green) was analysed by confocal microscopy using FV3000 software in 293T cells mock-infected or infected with BEV HY12 (MOI of 0.5) for 12 h. Cell nuclei were stained with 4 ‘,6-diamidino-2-phenylindole (DAPI) (blue). Scale bar, 10 μm. **F** 2C-His immobilised on anti-His immunomagnetic beads was incubated with lysates from 293T cells transfected with p65-Myc or Myc-vector plasmids. The bound proteins were detected by western blots using indicated antibodies.

To test this hypothesis, we co-transfected 293T cells with Flag-tagged 2C and His-tagged p65 plasmids and harvested for co-immunoprecipitated (Co-IP) assays. When exogenous Flag-tagged 2C proteins or His-tagged p65 were overexpressed in cells, they could precipitate each other (Figure 4A and B). This outcome indicates that 2C interacts with p65. Confocal microscopy was used to localise the 2C and p65 to confirm this interaction. Our results showed that 2C protein co-localised with p65 in the cytoplasm (Figure 4C).

We used Co-IP and confocal microscopy to corroborate this outcome further to examine the endogenous interaction between p65 and 2C in HY12-infected or mock-infected 293T cells. The endogenous interaction of 2C with p65 in the virus-infected cells was revealed (Figure 4D, E), confirming that 2C interacts with p65. The pull-down assay was used to determine whether 2C directly interacts with p65. The prokaryotic expressed His-2C protein labelled on anti-His magnetic beads successfully pulled down the Myc-tagged p65 (Figure 4F), suggesting that BEV 2C directly interact with p65.

### BEV 2C protein interacts with the IPT domain of p65 and inhibits the dimerisation of p65/p50

**Figure 5:**
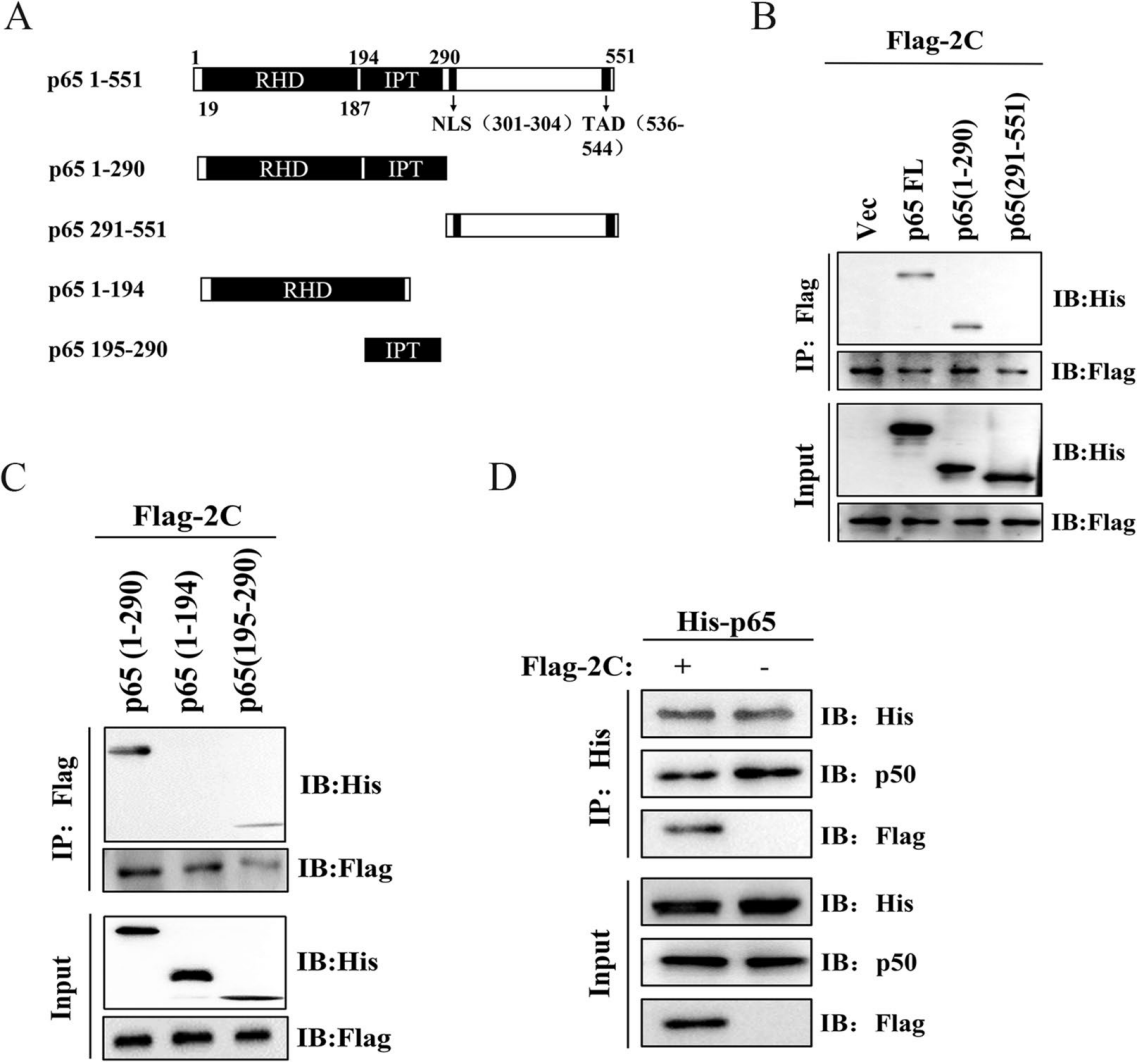
**2C protein interacts with the IPT domain of p65 and inhibits the dimerisation of p65/p50**. **A** Diagram of p65 truncations. Numbers indicated the amino acid position. **B** HY12 2C interacts with the 1–290 aa of p65. 293T cells co-transfected with Flag-tagged 2C (6.0 μg) and His-tagged p65 (6.0 μg) or His-tagged p65 truncates 1–290 aa (6.0 μg), 291–551 aa (6.0 μg) were used for immunoprecipitation using anti-Flag or anti-His antibody. Interactions between 2C and the truncated fragment of p65 were detected with immunoblotting. **C** HY12 2C interacts with the fragment of 194–290 aa of p65. 293T cells co-transfected with Flag-tagged 2C (6.0 μg) and His-tagged p65 truncates 1–290 aa (6.0 μg), 1–194 aa (6.0 μg), 195–290 aa (6.0 μg) were used for immunoprecipitation using anti-Flag or anti-His antibody. Interactions between 2C and the truncated fragment of p65 were detected with immunoblotting. **D** 2C inhibits p65/p50 dimerisation. 293T cells transfected with p65, p50, and 2C were harvested and analysed by coimmunoprecipitation and western blots using indicated antibodies.

To identify the region of p65 that interacts with 2C, we constructed truncated p65 mutants and expressed them in immunoprecipitation experiments (Figure 5A). Initially, the fragment 1–290 aa in p65 was shown to interact with 2C (Figure 5B). However, further dissection of the fragment (1–290 aa) revealed that the region spanning 195–290 aa in p65 interacted with 2C (Figure 5C).

As the region 195–290 aa corresponds to the IPT (Ig-like, plexins, transcription factors) domain of p65, which is involved in the p65 dimerisation with p50, we speculated that the interaction between 2C and p65 might also affect the dimerisation of p65 with p50. Subsequently, 293T cells co-transfected with either p65-His and 2C-Flag or p65-His and PCI-Flag were immunoprecipitated with anti-His. Our results show that 2C inhibited the association of p50 and p65-His (Figure 5D), suggesting that 2C reduces or disrupts p65/p50 dimer formation.

### N-terminal 118–121 aa of 2C interacts with IPT domain of p65

**Figure 6:**
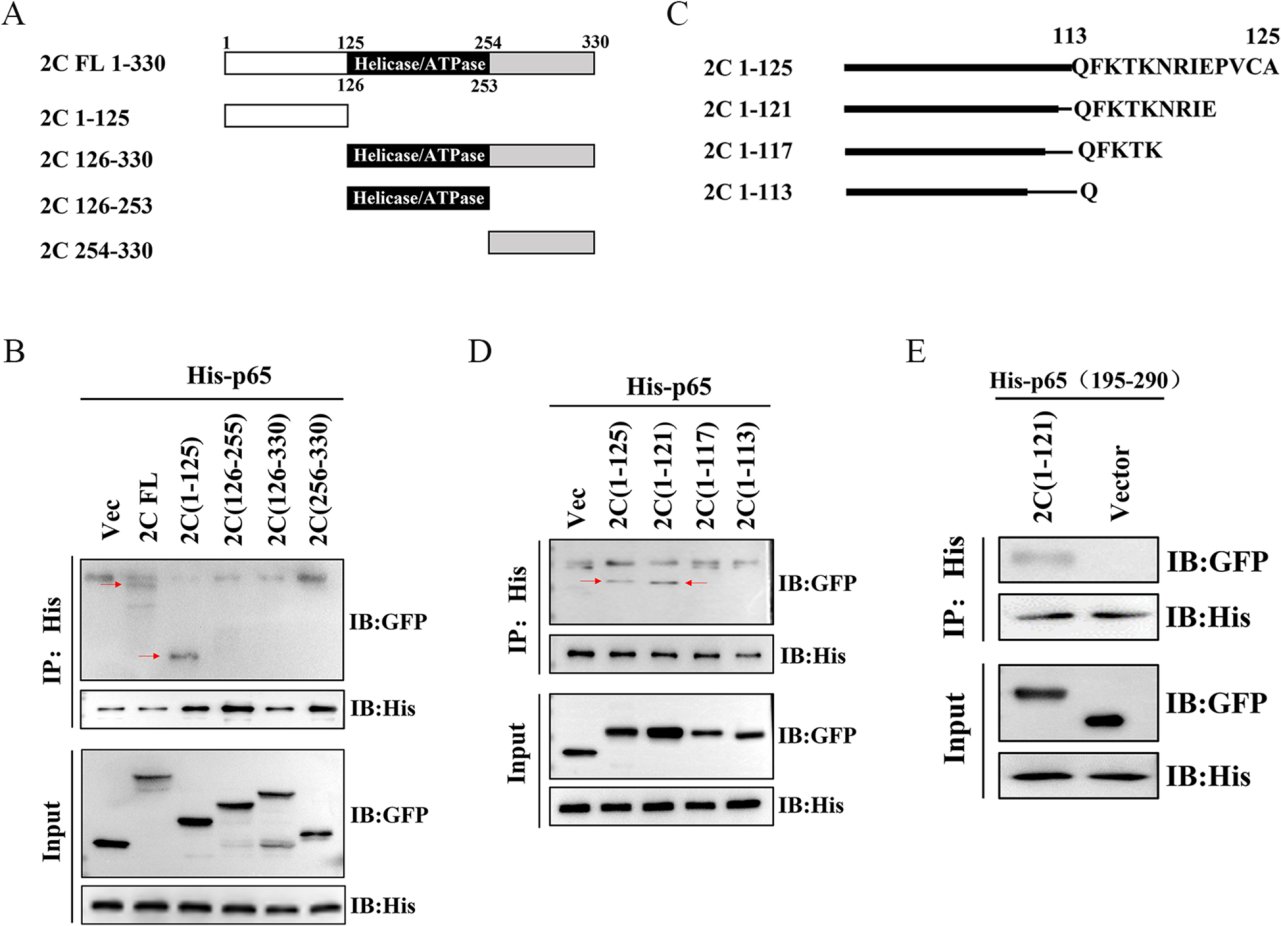
**N-terminal 118–121 aa of 2C interacted with the IPT domain of p65.** **A** Diagram of 2C truncations with the amino acid position numbers indicated. **B** N-terminal 125 aa of 2C interacted with p65. 293T cells co-transfected with His-tagged p65 (6.0 μg) and GFP-tagged 2C (6.0 μg) or GFP-tagged 2C truncates 1–125 aa (6.0 μg), 126–330 aa (6.0 μg), 126–255 aa (6.0 μg), 256–330 aa (6.0 μg) were used for immunoprecipitation using anti-GFP or anti-His antibody. Interactions between p65 and a truncated fragment of 2C were detected with immunoblotting. **C** The diagram shows the further dissection of 2C fragments 1–125, with the amino acid position numbers indicated. **D** 118–121 aa of 2C interacted with p65. 293T cells co-transfected with His-tagged p65 (6.0 μg) and GFP-tagged 2C truncates 1–125 aa (6.0 μg), 1–121 aa (6.0 μg), 1–117 aa (6.0 μg), 1–113 aa (6.0 μg) were used for immunoprecipitation using anti-GFP or anti-His antibody. Interactions between p65 and a truncated fragment of 2C were detected with immunoblotting. **E** 118–121 aa of 2C interacted with IPT domain of p65. 293T cells co-transfected with His-tagged IPT (6.0 μg) and GFP-tagged 2C 1–121 (6.0 μg) or GFP-vector (6.0 μg) were used for immunoprecipitation using anti-GFP or anti-His antibody. Interactions between the IPT domain of p65 and 1-121aa of 2C were detected with immunoblotting.

To delineate the minimal region of 2C responsible for interacting with p65, a series of truncated 2C mutants were constructed based on 2C domain predictions from the InterPro website (Figure 6A). Cells transfected with 2C mutants were harvested for immunoprecipitation assay. Fragment spanning 1–125 aa in 2C were found to bind with p65 (Figure 6B) specifically.

Deletion mutants, including 1–125 aa, 1–121 aa, 1–117 aa, and 1–113 aa in 2C, were generated (Figure 6C) to map further the domain of how 2C interacts with the IPT domain of p65. The deletion mutants 1–125 aa and 1–121 aa co-immunoprecipitated the p65, whereas the deletion mutants 1–117 aa and 1–113 aa showed no interaction with p65 (Figure 6D).

These results demonstrated that the four amino acids (118–121) of 2C interacted with the IPT domain of p65. The results of the Co-IP analysis showed that IPT was able to successfully extract GFP-2C (1–121) from 293 cells that were co-transfected with pcDNA-his-IPT and either pEGFP-2C (1–121) or pEGFP-vector (Figure 6E).

The above results demonstrate that N-terminal 121 aa of 2C interacted with the IPT domain of p65, and the four amino acids (118–121) are the binding site.

### N-terminal 1–121 aa of 2C inhibits p65 phosphorylation and nuclear translocation to promote viral replication

**Figure 7:**
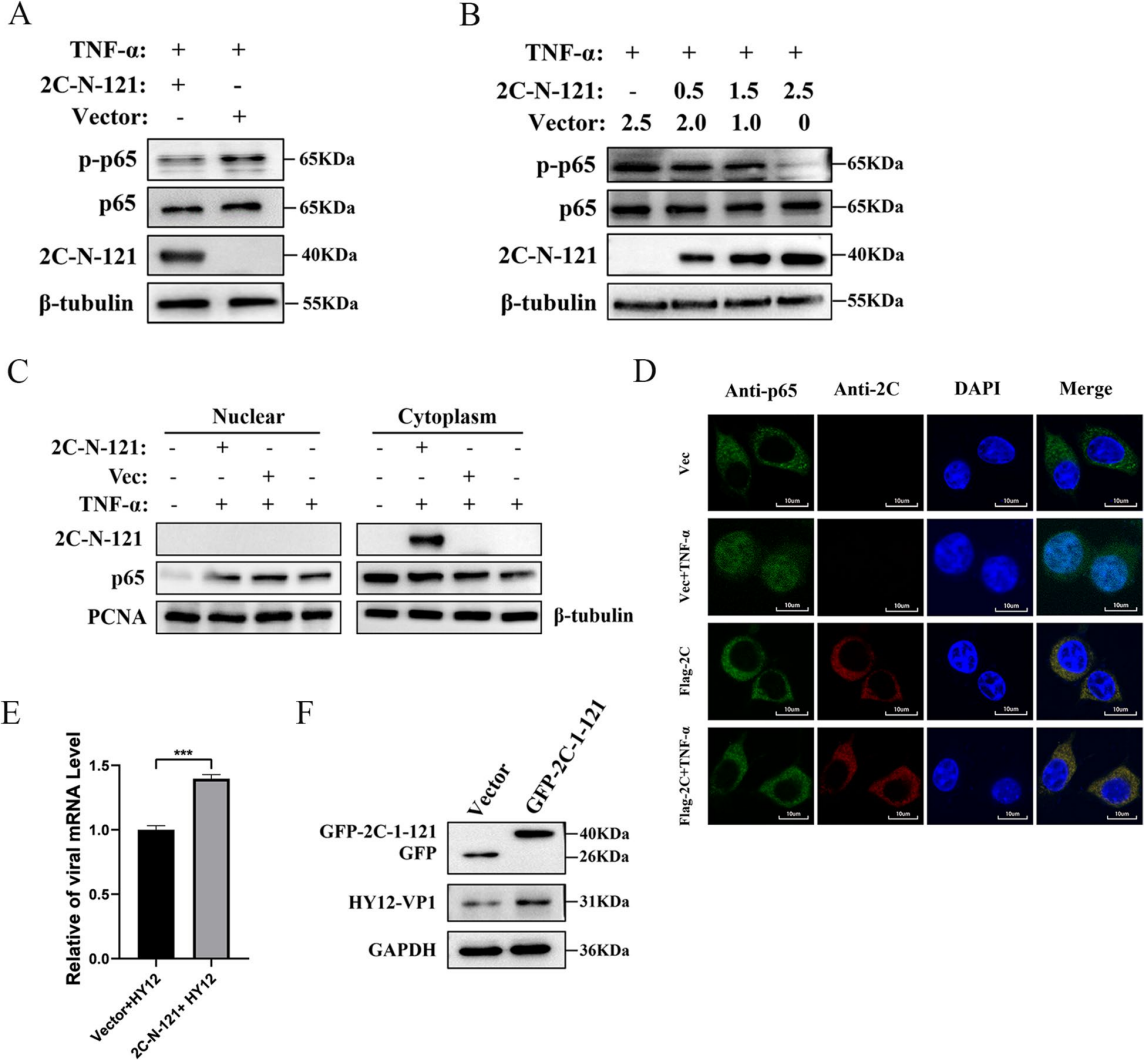
**N-terminal 121 aa of 2C inhibits P65 phosphorylation and nuclear translocation to promote viral replication.** **A**, **B** N-terminal 121 aa of 2C inhibits P65 phosphorylation. **A** 293T cells transfected with pEGFP-2C-1–121 (2.5 μg) or pEGFP-vector (2.5 μg) for 48 h were treated with TNF-α for 20 min. Western blot analyses detected expressions of GFP-2C-1–121, p-p65, p65, and β-Tubulin protein. **B** 293T cells transfected with pEGFP-2C-1–121 or pEGFP-vector for 48 h were treated with TNF-α for 20 min. Western blot analyses detected expressions of GFP-2C-1–121, p-p65, p65, and β-Tubulin protein. **C**, **D** N-terminal 125 aa of 2C inhibits nuclear translocation of p65. **C** 293T cells transfected with pEGFP-2C-1–121 (2.5 μg) or pEGFP-vector (2.5 μg) for 48 h were treated with or without TNF-α at a 20 ng/mL concentration for 20 min. Cell lysates were then separated into cytoplasmic and nuclear fractions. Western blot analyses with anti-p65 Ab analysed cytoplasmic and nuclear proteins to reveal the localisation of NF-κB subunits. Nucleus-specific anti-PCNA Ab and cytoplasmic-specific anti-tubulin or GAPDH Ab were used as controls. **D** Localisation analysis of p65 was performed by confocal microscopy using FV3000 software. 293T cells were treated in the same way as described in **C**. A laser confocal microscope was used to localise p65 (green) HY12-VP1 (red). Cell nuclei were stained with 4ʹ,6-diamidino-2-phenylindole (DAPI) (blue). Scale bar, 10 μm. **E**, **F** N-terminal 121 aa of 2C promotes viral replication. **E** 293T cells were transfected with pEGFP-2C-1–121 (2.5 μg) or pEGFP-vector (2.5 μg) for 24 h before they were infected by HY12 viruses for 24 h. The expression level of viral VP1 mRNA was detected by qPCR. GAPDH as an internal control. **F** 293T cells transfected with either pEGFP-2C-1–121 (2.5 μg) or pEGFP-vector (2.5 μg) for 24 h were infected by BEV HY12 for 24 h. Expressions of GFP-2C-1–121, GFP, HY12-VP1, and GAPDH protein were detected by western blotting.

As demonstrated, the 1–121 aa of 2C interacts with the IPT domain of p65 to decrease the formation of p65 and p50 dimers. We theorised that this effect might inhibit the phosphorylation of p65 and its nuclear translocation, the key step in activating the NF-κB signalling pathway. We treated 293T cells transfected with pEGFP-2C-1-121 with TNF-α and used a western blot analysis to detect p-p65 and p65 to substantiate this theory. Compared with the empty vector control, the expression level of p-p65 was significantly lower in cells transfected with the 2C 1–121 plasmid following TNF-α treatment (Figure 7A). Moreover, the 2C 1–121 inhibition of p65 phosphorylation was dose-dependent (Figure 7B).

To determine the effect of 2C 1–121 on p65 translocation, cells were transfected with a plasmid harbouring 2C 1–121 aa or with empty vector. After treatment, with or without TNF-α, the cell lysates were separated into cytoplasmic and nuclear fractions for immunoblotting analysis. The expression level of p65 from cells transfected with the 2C 1–121 plasmid was significantly lower than those transfected with empty vectors following TNF-α stimulation.

The expression of p65 in the cytoplasm was significantly higher in cells transfected with the 2C 1–121 plasmid than those transfected with empty vectors (Figure 7C). This outcome suggests that 2C 1–121 inhibits the translocation of TNF-α-triggered p65 from the cytoplasm to the nucleus. Confocal microscopy revealed similar results with respect to the expression location of p65. Compared to the empty vector control group, 2C 1–121 inhibited the TNF-α-triggered nuclear translocation of p65 (Figure 7D).

To determine the biological effect of 2C 1–121 on HY12 virus replication, 293T cells transfected with 2C 1–121 plasmid or empty vector plasmid were infected with the HY12 virus. The mRNA and protein levels of HY12-VP1 were analysed separately by RT-qPCR and western blotting. The mRNA and protein levels of VP1 were significantly increased in the infected cells overexpressing 2C 1–121 (Figure 7E and F). These results demonstrated that 2C 1–121 was essential for BEV replication and promote BEV replication.

In summary, the above results demonstrated the role of BEV 2C, defined its domain in promoting virus replication, and elucidated the mechanism by which 2C 1–121 promotes viral replication by inhibiting the activation of NF-κB in host cells.

### Inhibition activation of the NF-κB signalling pathway by 2C protein revealed in cells infected by EV-F

**Figure 8:**
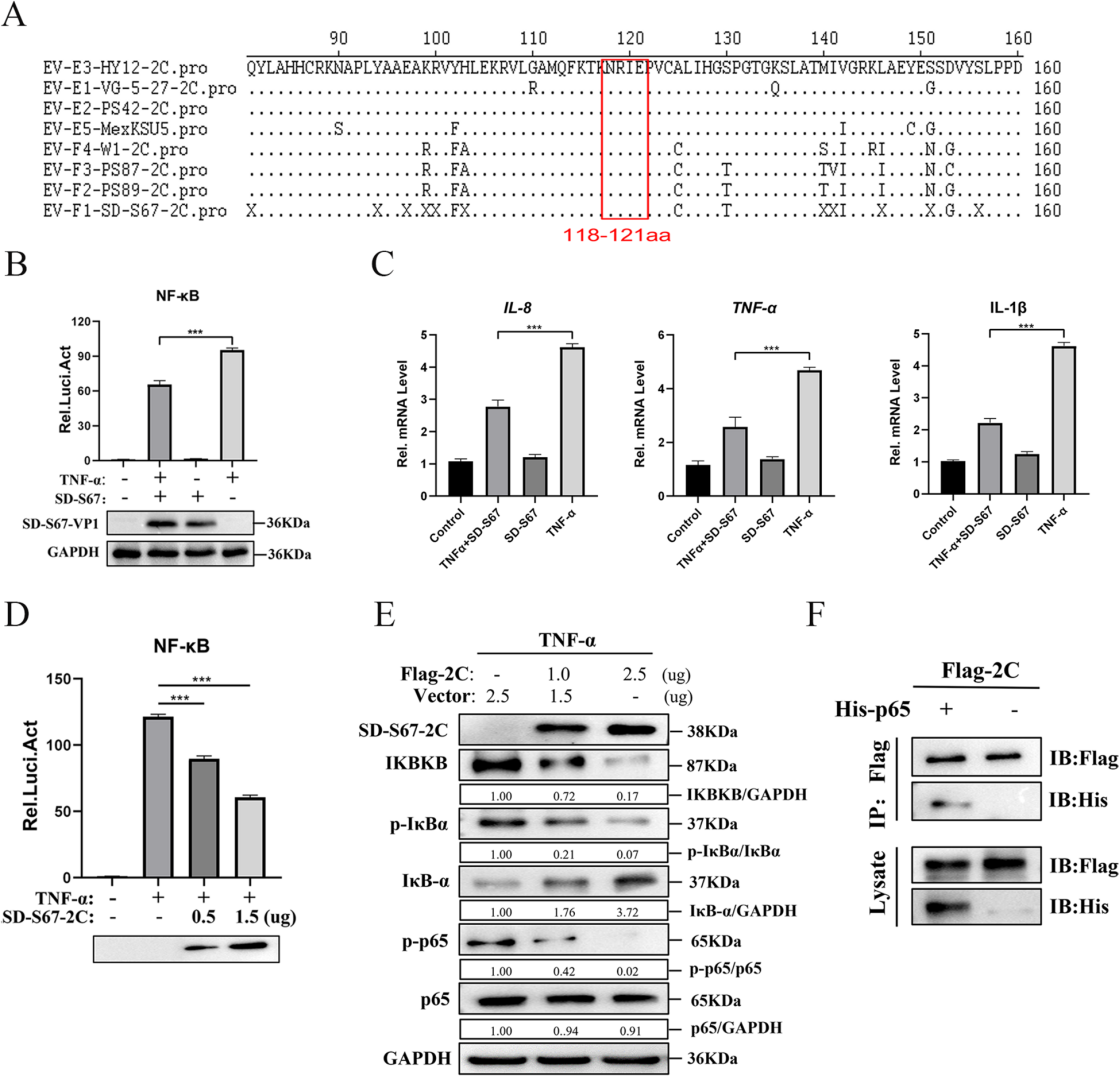
**BEV 2C protein inhibits the TNF-α-triggered NF-κB activation.** **A** Sequence alignment analysis of representative EV-F and EV-E strains using MegAlign software. **B** 293T cells were co-transfected with NF-κB-Luc reporter plasmid (0.4 μg) and pRL-SV40 plasmid (0.08 μg). Twenty-four hours post-transfection, the cells infected by EV-F SD-S67 (MOI of 0.5) for 10 h or mock-infected were treated with TNF-α (20 ng/mL) for 6 h and harvested for luciferase reporter gene assays. **C** Expression levels of mRNA for IL-8, IL-1β, and TNF-α were measured by qPCR with GAPDH as an internal reference gene. **D** 293T cells were transfected with SD-S67 2C expressing plasmid at different concentrations (1.0 or 2.5 μg) along with pNF-κB-Fluc (0.4 μg) and pRL-TK (0.08 μg). Twenty-four hours post-transfection, the luciferase assay and immunoblotting analysis were performed after the cells were treated with TNF-α (20 ng/mL) for 6 h. **E** 293T cells transfected with PCI-neo (Vector) (2.5, 1.5, 0 μg) and PCI-neo-2C (0, 1.0, 2.5 μg) for 48 h were treated with TNF-α (20 ng/mL) for 20 min. The expressions of Flag-2C, IΚBΚB, IκBα, p-IκBα, p-p65, p65 and GAPDH protein were detected by western blot analyses. **F** 293T cells co-transfected with Flag-tagged 2C (6.0 μg) and His-tagged p65 (6.0 μg) or His-vector were immunoprecipitated using anti-Flag or anti-His antibody. Interactions between 2C and p65 were detected with immunoblotting.

Sequence alignment analysis of the 2C protein from EV-F and EV-E representative strains was performed to investigate whether the above conclusions are generalised in bovine enteroviruses. The region of 2C (118–121 aa) for interaction between HY12-2C and p65 was highly conserved among EV-F and EV-E enterovirus (Figure 8A).

To explore whether the inhibition of the NF-κB signalling pathway by 2C protein is a general trend, a representative EV-F strain, SD-S67, was used to examine this idea. The luciferase activity of SD-S67-infected cells was significantly lower than that of mock-infected cells (Figure 8B), indicating that SD-S67 virus infection leads to the inhibition of the NF-κB signalling pathway.

Further detection of the transcriptional levels of the inflammatory cytokines IL-8, IL-1β, and TNF-α using quantitative RT-PCR (RT-qPCR) demonstrated that SD-S67 significantly inhibited the TNF-α-induced mRNA expression levels of the IL-8, IL-1β, and TNF-α genes (Figure 8C). This finding suggests that SD-S67 is involved in regulating endogenous NF-κB signalling.

The above results demonstrate that EV-F SD-S67 significantly inhibits the TNF-α-triggered NF-κB signalling pathway.

Furthermore, a dual-fluorescence reporter gene assay was performed to determine whether the 2C protein of the SD-S67 strain inhibits the activation of TNF-α-triggered NF-κB. The inhibition of SD-S67-2C on the NF-κB promoter was shown as dose-dependent (Figure 8D). In addition, western blot results indicated that the 2C protein of SD-S67 also inhibited the expression of the IKBKB protein, phosphorylation and degradation of IKB-α and activated the NF-κB signalling pathway (Figure 8E). Furthermore, the Co-IP assay demonstrated the interaction of the 2C protein encoded by the SD-S67 virus with the p65 protein (Figure 8F).

The above results demonstrated the same inhibition of NF-κB activation by EV-F-2C (SD-S67) and the underlying mechanism to EV-E 2C protein, suggesting the generality of EV-F- or EV-E-encoded 2C on NF-κB activation.

## Discussion

The innate immune system is a highly conserved signalling network that acts as the first line of defence for the host against viral infections. When host senses the conserved viral structural components, PAMPs, through PRRs, its innate immune response is initiated [40–42]. This leads to the expression of downstream antiviral effector proteins, such as type I interferons and pro-inflammatory cytokines [41, 43].

Many viruses have evolved mechanisms that modulate the NF-κB pathway to evade the host immune response. For example, the Hantavirus N protein inhibits TNF-α-induced NF-κB activation by sequestering p65 in the cytoplasm by binding to nuclear input proteins [44]; the Herpes Simplex Virus 1 UL2 inhibits the TNF-α-Mediated NF-κB activity by interacting with p65/p50 [45], and the Rotavirus NSP1 protein inhibits NF-κB activation by inducing proteasome-dependent degradation of proteins containing β-transduction protein repeats [46, 47]. In this study, we report that BEV 2C protein inhibits the activation of the key proteins downstream of the NF-κB signalling pathway by reducing the expression of IKBKB. Moreover, the 2C protein can also directly interact with the transcription factor p65 to inhibit its nuclear translocation and phosphorylation, thereby inhibiting the activation of NF-κB induced by viruses and TNF-α.

Enterovirus is a single-stranded positive-stranded picornavirus. After entering the host cells, the virus immediately employs host machinery and the released viral genome to synthesise the large precursor protein. The precursor protein was cleaved into four structural proteins (VP1, VP2, VP3, and VP4) and seven non-structural proteins (2A, 2B, 2C, 3A, 3B, 3C, and 3D) [48]. Subsequently, the enterovirus infection induced a surge of pro-inflammatory cytokines, resulting in elevated levels of pro-inflammatory and inflammatory cytokines, including TNF-α [39]. These cytokines were used by the host to counteract the viruses. TNF-α, one of the most pleiotropic pro-inflammatory cytokines, triggers numerous cellular responses, including cytotoxicity, antiviral activity, proliferation, and transcriptional regulation of various genes [34].

Previous studies have demonstrated that enteroviruses activate antiviral signalling pathways during the early stages of infection, thus interfering with increased expression levels of stimulus genes and pro-inflammatory cytokines [49], particularly the NF-κB signalling pathway. Tung et al. demonstrated a time-dependent increase in NF-κB-regulated luciferase activity in mouse smooth muscle cells within half an hour of EV-A71 infection. They found that the SK-N-SH cells infected by EV-A71 triggers the translocation of NF-κB (p65) into the nucleus and the degradation of IκBα at 1 hpi [50, 51].

Based on previous studies and our accumulative results, we hypothesised that enteroviruses have developed certain mechanisms to inhibit NF-κB activation by modulating the antiviral response in the later stages of infection. Moreover, our experimental results demonstrated that BEV infection inhibits TNF-α-induced NF-κB (p65) nuclear translocation and the activation of NF-κB transcription. These findings suggested that TNF-α-induced NF-κB activation is inhibited in E enterovirus-infected cells. Therefore, this result may be an important mechanism by which enteroviruses modulate host immune responses.

Several other enteroviruses, including coxsackievirus B3, foot-and-mouth disease virus, and EV-A71, can hinder the activation of host cell NF-κB through viral proteins. For example, the 3C protein of coxsackievirus B3 inhibits the transactivation of NF-κB by cleaving IκBα [52]. Similarly, the 2C protein of EV-A71 reduces the phosphorylation of IKBKB by interacting with IKBKB and then inhibits TNF-α-mediated NF-κB activation [52]. The L protein of foot and mouth disease (FMD) virus inhibits p65 entry into the nucleus by degrading p50/p65 dimers, thereby inhibiting the activation of NF-κB [32]. In addition, the 3A protein of poliovirus inhibits NF-κB signalling by eliminating TNFR on the cell surface [30].

In conjunction with the above, our study found that the BEV HY12 can also suppress the signal transduction of NF-κB. However, the BEV achieved this by employing a new strategy to inhibit NF-κB, using the BEV’s non-structural protein 2C as an antagonist of NF-κB activation. Notably, the 2C protein has been reported to have ATPase and membrane-bound activity. It acts as an NTPase and guides replication complexes to the cell membrane [53]. Furthermore, the 2C protein of EV-A71 and CV-A16 have been shown to possess ATP-dependent RNA helicase and ATP-independent chaperoning activities, which are critical for viral RNA replication [54]. Sequence analysis indicated that the 2C protein was the most conserved protein among all picornaviruses, with the 2C of EV-A71 having 97.5% and 63.3% homology with 2C of CA-16 and poliovirus. In our study, we found that the 2C protein of HY12 enterovirus had a sequence identity of only 67.5% with that of EV-A71, which was relatively conserved but different. This result could explain why the immune escape mechanism of BEV differs from that of EV-A71.

Research on enterovirus 2C has recently increased due to the highly conserved nature of the protein’s multiple functions. Zheng et al. showed that EV-71 2C protein inhibits TNF-α-mediated activation of NF-κB by suppressing IκB Kinase-β phosphorylation [33]. Furthermore, Tang et al. demonstrated that Reticulon 3 (RTN3) binds to the 2C protein of EV-A71 and is required for viral replication [15]. Recently, Ji et al. revealed that enterovirus 2C protein suppresses IKKα phosphorylation by recruiting IKKβ and IKKα into viral inclusion bodies [55]. Our results showed that the overexpression of 2C reduced the mRNA and protein expression of IKBKB, which inhibited the phosphorylation and degradation of IκB-α, a key protein downstream of NF-κB. Subsequently, it blocked the phosphorylation and nuclear translocation of nuclear transcription factor p65, thus inhibiting the activation of NF-κB. This blocking is an evolved and new strategy that BEV has developed to modulate the innate immune response.

It is widely known that p65 directly influences NF-κB activation within the innate immune signalling pathway. Upon stimulation, it is activated and moves into the nucleus to modulate the transcription of various genes, including inflammatory factors, chemokines [56], and cytokines. This process is directly or indirectly linked to antiviral responses and helps inhibit viral replication [57]. However, viruses employ different strategies to evade the host’s immune response. Moreover, many viruses use the encoded proteins to interact directly with p65, thus affecting its activation. For instance, Jiao et al. demonstrated that the adenovirus E1A protein prevents the phosphorylation of p65 by directly binding to p65 [58]. Jobe et al. reported that the respiratory syncytial virus (RSV) NS1 and NS2 proteins directly interact with p65 to suppress the transcriptional activity of p65 and inhibit the activation of the NF-κB pathway, thereby evading the host’s immune response [59].

We employed the Co-IP and protein pull-down assays to explore the functions and underlying mechanism of 2C of BEV in regulating host innate immunity and virus replication. We found that the 2C protein of HY12 interacts with p65 directly. These results indicate that 2C protein not only inhibits the activation of NF-κB by reducing the expression of IKBKB but also directly interacts with p65 to regulate NF-κB signal transduction. Similarly, inhibition of the NF-κB signalling pathway by EV-F 2C protein was demonstrated. These results further suggested that the 2C protein of BEV inhibited the NF-κB signalling using a general pattern. Furthermore, we truncated the p65 and 2C proteins, mapped the key sites for interaction, and revealed that the four amino acids (118–121 aa) of 2C interact with the IPT domain of p65. This finding is especially crucial in searching for new drug targets and developing novel vaccines.

**Figure 9:**
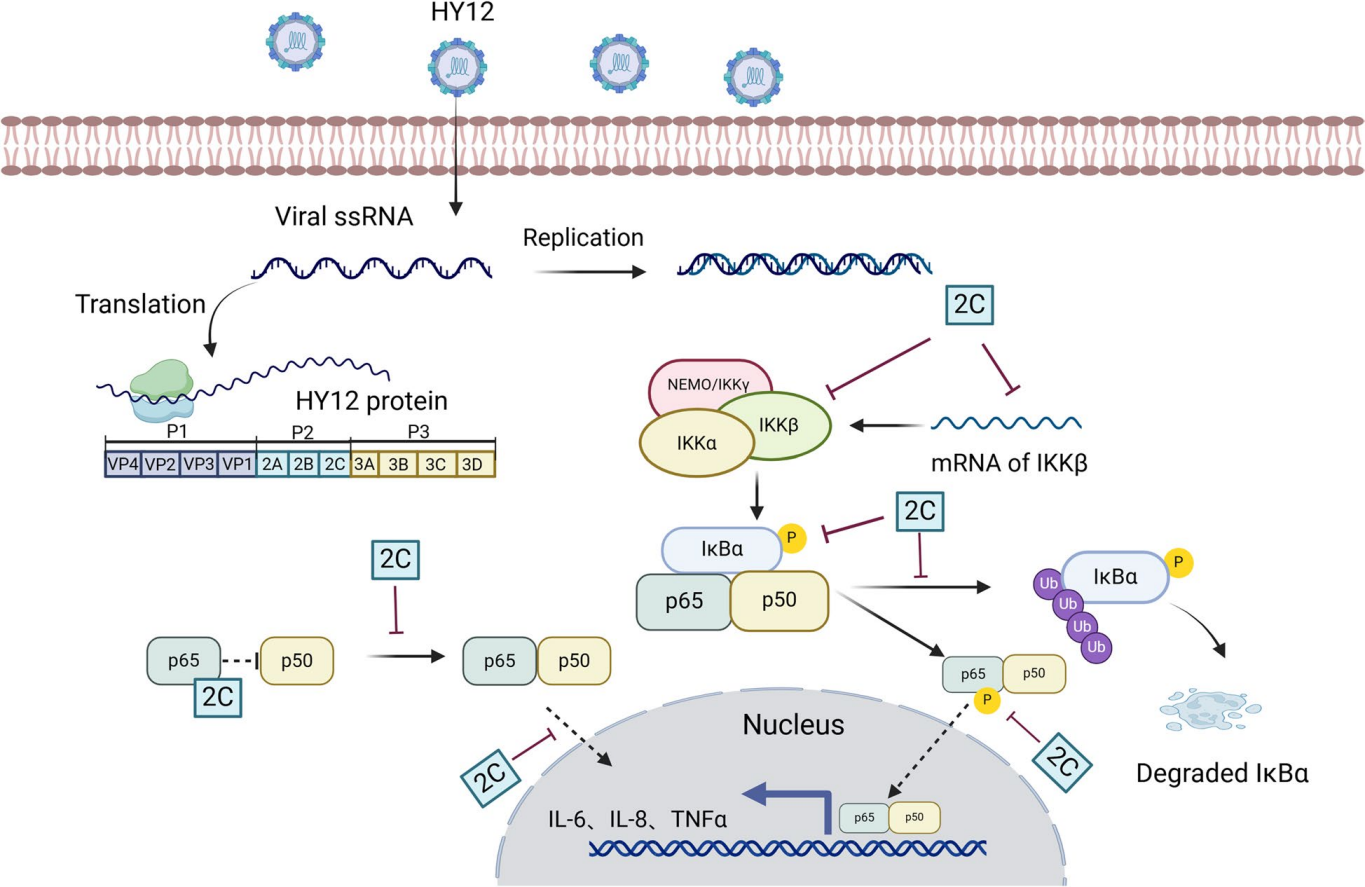
**Schematic diagram of the proposed mechanism for the HY12 virus to promote its replication**. After HY12 viruses enter the cells, the viral genome released from the virus particle was used as an mRNA to translate the precursor polyproteins, which were processed into virus structural and non-structural proteins. The BEV 2C, on the one hand, inhibits the expression of IKKβ, which further blocks the phosphorylation and degradation of the downstream protein IκBα, and ultimately inhibits the phosphorylation and nuclear translocation of p65, and decreases the expression of inflammatory factors IL-8, IL-1β, TNF-α. On the other band, 2C 1-125aa directly interacted with p65 inhibited the dimerisation of p65/p50, blocked the phosphorylation of p65 and nucleus entry, and reduced the expression of inflammatory factor IL-8, IL-1β, TNF-α, thus promoting its replications.

In conclusion, we demonstrated that BEV inhibits the activation of NF-κB to promote virus replication. Using two strategies, we unveiled the novel mechanisms for BEV to escape the host immune response. Based on our findings, we proposed the model for HY12 viruses to inhibit the host NF-κB immune signalling pathway to promote viral replication (Figure 9).

One strategy employed by HY12 viruses, upon entering cells, is to use the 2C protein to suppress the expression of IKBKB. This effect, in turn, blocks the activation of downstream proteins, including the phosphorylation of IκBα and p65. Another strategy is that HY12 enterovirus uses its encoded 2C protein to interact directly with p65, blocking the dimerisation of p65/p50. This method inhibits the phosphorylation and nuclear translocation of p65, thus inhibiting the NF-κB signalling pathway. Since 2C is a viral factor involved in innate immune evasion during EV infection, future studies will focus on exploring the antiviral drugs that affect the interaction of 2C with the IPT domain of p65.

## Data Availability

The datasets used during the current study are available from the corresponding author upon reasonable request.

## References

[1] Lefkowitz EJ, Dempsey DM, Hendrickson RC, Orton RJ, Siddell SG, Smith DB (2018) Virus taxonomy: the database of the International Committee on Taxonomy of Viruses (ICTV). Nucleic Acids Res 46:D708–D717 29040670 10.1093/nar/gkx932 PMC5753373

[2] Zhu L, Xing Z, Gai X, Li S, San Z, Wang X (2014) Identification of a novel enterovirus E isolates HY12 from cattle with severe respiratory and enteric diseases. PLoS One 9:e97730 24830424 10.1371/journal.pone.0097730 PMC4022658

[3] McMinn PC (2022) An overview of the evolution of enterovirus 71 and its clinical and public health significance. FEMS Microbiol Rev 26:91–107 10.1111/j.1574-6976.2002.tb00601.x 12007645

[4] Lu HH, Li X, Cuconati A, Wimmer E (1995) Analysis of picornavirus 2A(pro) proteins: separation of proteinase from translation and replication functions. J Virol 69:7445–7452 7494250 10.1128/jvi.69.12.7445-7452.1995 PMC189682

[5] Fujita K, Krishnakumar SS, Franco D, Paul AV, London E, Wimmer E (2007) Membrane topography of the hydrophobic anchor sequence of poliovirus 3A and 3AB proteins and the functional effect of 3A/3AB membrane association upon RNA replication. Biochemistry 46:5185–5199 17417822 10.1021/bi6024758 PMC2519882

[6] Norder H, De Palma AM, Selisko B, Costenaro L, Papageorgiou N, Arnan C, Coutard B, Lantez V, De Lamballerie X, Baronti C, Solà M, Tan J, Neyts J, Canard B, Coll M, Gorbalenya AE, Hilgenfeld R (2011) Picornavirus non-structural proteins as targets for new anti-virals with broad activity. Antiviral Res 89:204–218 21236302 10.1016/j.antiviral.2010.12.007

[7] Banerjee R, Weidman MK, Echeverri A, Kundu P, Dasgupta A (2004) Regulation of poliovirus 3C protease by the 2C polypeptide. J Virol 78:9243–9256 15308719 10.1128/JVI.78.17.9243-9256.2004 PMC506913

[8] Li JP, Baltimore D (1990) An intragenic revertant of a poliovirus 2C mutant has an uncoating defect. J Virol 64:1102–1107 2154595 10.1128/jvi.64.3.1102-1107.1990 PMC249223

[9] Teterina NL, Gorbalenya AE, Egger D, Bienz K, Ehrenfeld E (1997) Poliovirus 2C protein determinants of membrane binding and rearrangements in mammalian cells. J Virol 71:8962–8972 9371552 10.1128/jvi.71.12.8962-8972.1997 PMC230196

[10] Aldabe R, Carrasco L (1995) Induction of membrane proliferation by poliovirus proteins 2C and 2BC. Biochem Biophys Res Commun 206:64–76 7818552 10.1006/bbrc.1995.1010

[11] Banerjee R, Echeverri A, Dasgupta A (1997) Poliovirus-encoded 2C polypeptide specifically binds to the 3’-terminal sequences of viral negative-strand RNA. J Virol 71:9570–9578 9371621 10.1128/jvi.71.12.9570-9578.1997 PMC230265

[12] Teterina NL, Levenson E, Rinaudo MS, Egger D, Bienz K, Gorbalenya AE, Ehrenfeld E (2006) Evidence for functional protein interactions required for poliovirus RNA replication. J Virol 80:5327–5337 16699013 10.1128/JVI.02684-05 PMC1472133

[13] Wang SH, Wang K, Zhao K, Hua SC, Du J (2020) The structure, function, and mechanisms of action of enterovirus non-structural protein 2C. Front Microbiol 11:615965 33381104 10.3389/fmicb.2020.615965 PMC7767853

[14] Xia H, Wang P, Wang GC, Yang J, Sun X, Wu W, Qiu Y, Shu T, Zhao X, Yin L, Qin CF, Hu Y, Zhou X (2015) Human enterovirus nonstructural protein 2CATPase functions as both an RNA helicase and ATP-independent RNA chaperone. PLoS Pathog 11:e1005067 26218680 10.1371/journal.ppat.1005067 PMC4517893

[15] Tang WF, Yang SY, Wu BW, Jheng JR, Chen YL, Shih CH, Lin KH, Lai HC, Tang P, Horng JT (2007) Reticulon 3 binds the 2C protein of enterovirus 71 and is required for viral replication. J Biol Chem 282:5888–5898 17182608 10.1074/jbc.M611145200

[16] Yeager C, Carter G, Gohara DW, Yennawar NH, Enemark EJ, Arnold JJ, Cameron CE (2022) Enteroviral 2C protein is an RNA-stimulated ATPase and uses a two-step mechanism for binding to RNA and ATP. Nucleic Acids Res 50:11775–11798 36399514 10.1093/nar/gkac1054 PMC9723501

[17] Pahl HL (1999) Activators and target genes of Rel/NF-κB transcription factors. Oncogene 18:6853–6866 10602461 10.1038/sj.onc.1203239

[18] Mettelman RC, Allen EK, Thomas PG (2022) Mucosal immune responses to infection and vaccination in the respiratory tract. Immunity 55:749–780 35545027 10.1016/j.immuni.2022.04.013 PMC9087965

[19] Karin M, Ben-Neriah Y (2000) Phosphorylation meets ubiquitination: the control of NF-κB activity. Annu Rev Immunol 18:621–663 10837071 10.1146/annurev.immunol.18.1.621

[20] Scheidereit C (2006) IκB kinase complexes: gateways to NF-κB activation and transcription. Oncogene 25:6685–6705 17072322 10.1038/sj.onc.1209934

[21] Hayden MS, Ghosh S (2011) NF-κB in immunobiology. Cell Res 21:223–244 21243012 10.1038/cr.2011.13 PMC3193440

[22] Romero N, Favoreel HW (2021) Pseudorabies virus infection triggers NF-κB activation via the DNA damage response but actively inhibits NF-κB-dependent gene expression. J Virol 95:e0166621 34613805 10.1128/JVI.01666-21 PMC8610585

[23] Song K, Li S (2021) The role of ubiquitination in NF-κB signaling during virus infection. Viruses 13:145 33498196 10.3390/v13020145 PMC7908985

[24] Santoro MG, Rossi A, Amici C (2003) NF-κB and virus infection: who controls whom. Embo J 22:2552–2560 12773372 10.1093/emboj/cdg267 PMC156764

[25] Park J, Kang W, Ryu SW, Kim WI, Chang DY, Lee DH, Park DY, Choi YH, Choi K, Shin EC, Choi C (2012) Hepatitis C virus infection enhances TNFα-induced cell death via suppression of NF-κB. Hepatology 56:831–840 22430873 10.1002/hep.25726

[26] Tong M, Yi L, Sun N, Cheng Y, Cao Z, Wang J, Li S, Lin P, Sun Y, Cheng S (2017) Quantitative analysis of cellular proteome alterations in CDV-infected mink lung epithelial cells. Front Microbiol 8:2564 29312244 10.3389/fmicb.2017.02564 PMC5743685

[27] Ludwig S, Planz O (2008) Influenza viruses and the NF-κB signaling pathway-towards a novel concept of antiviral therapy. Biol Chem 389:1307–1312 18713017 10.1515/BC.2008.148

[28] Yang J, Li S, Feng T, Zhang X, Yang F, Cao W, Chen H, Liu H, Zhang K, Zhu Z, Zheng H (2021) African swine fever virus F317L protein inhibits NF-κB activation to evade host immune response and promote viral replication. mSphere 6:e0065821 34668754 10.1128/mSphere.00658-21 PMC8527992

[29] Diel DG, Luo S, Delhon G, Peng Y, Flores EF, Rock DL (2011) A nuclear inhibitor of NF-κB encoded by a poxvirus. J Virol 85:264–275 20980501 10.1128/JVI.01149-10 PMC3014193

[30] Neznanov N, Kondratova A, Chumakov KM, Angres B, Zhumabayeva B, Agol VI, Gudkov AV (2001) Poliovirus protein 3A inhibits tumor necrosis factor (TNF)-induced apoptosis by eliminating the TNF receptor from the cell surface. J Virol 75:10409–10420 11581409 10.1128/JVI.75.21.10409-10420.2001 PMC114615

[31] Zaragoza C, Saura M, Padalko EY, Lopez-Rivera E, Lizarbe TR, Lamas S, Lowenstein CJ (2006) Viral protease cleavage of inhibitor of κBα triggers host cell apoptosis. Proc Natl Acad Sci USA 103:19051–19056 17138672 10.1073/pnas.0606019103 PMC1748175

[32] de Los ST, Diaz-San Segundo F, Grubman MJ (2007) Degradation of nuclear factor κB during foot-and-mouth disease virus infection. J Virol 81:12803–12815 17881445 10.1128/JVI.01467-07 PMC2169123

[33] Zheng Z, Li H, Zhang Z, Meng J, Mao D, Bai B, Lu B, Mao P, Hu Q, Wang H (2011) Enterovirus 71 2C protein inhibits TNF-α-mediated activation of NF-κB by suppressing IκB kinase β phosphorylation. J Immunol 187:2202–2212 21810613 10.4049/jimmunol.1100285

[34] Lin TY, Hsia SH, Huang YC, Wu CT, Chang LY (2003) Proinflammatory cytokine reactions in enterovirus 71 infections of the central nervous system. Clin Infect Dis 36:269–274 12539066 10.1086/345905

[35] Huang PN, Shih SR (2014) Update on enterovirus 71 infection. Curr Opin Virol 5:98–104 24727707 10.1016/j.coviro.2014.03.007

[36] Wang Y, Hu J, Zhang F, Chang X, Tursun G, Zhang Q, Hu H, Wang X (2022) Inhibition of enterovirus HY12 replication by activating host’s NLRP3 signaling pathway. Chin J Vet Sci 42:1387–1392

[37] Wulff NH, Tzatzaris M, Young PJ (2012) Monte Carlo simulation of the Spearman-Kaerber TCID50. J Clin Bioinforma 2:5 22330733 10.1186/2043-9113-2-5 PMC3331834

[38] Schmittgen TD, Livak KJ (2008) Analyzing real-time PCR data by the comparative C(T) method. Nat Protoc 3:1101–1108 18546601 10.1038/nprot.2008.73

[39] Chang X, Guo Y, Zhang Q, Zheng X, Cui X, Hu J, Zhang Z, Zhang F, Wang X (2024) GRP78 recognizes EV-F 3D protein and activates NF-κB to repress virus replication by interacting with CHUK/IKBKB. J Virol 98:e0026824 38775480 10.1128/jvi.00268-24 PMC11237669

[40] Hu MM, Shu HB (2020) Innate immune response to cytoplasmic DNA: mechanisms and diseases. Annu Rev Immunol 38:79–98 31800327 10.1146/annurev-immunol-070119-115052

[41] Yang Q, Shu HB (2020) Deciphering the pathways to antiviral innate immunity and inflammation. Adv Immunol 145:1–36 32081195 10.1016/bs.ai.2019.11.001

[42] Wu J, Chen ZJ (2014) Innate immune sensing and signaling of cytosolic nucleic acids. Annu Rev Immunol 32:461–488 24655297 10.1146/annurev-immunol-032713-120156

[43] Amarante-Mendes GP, Adjemian S, Branco LM, Zanetti LC, Weinlich R, Bortoluci KR (2018) Pattern recognition receptors and the host cell death molecular machinery. Front Immunol 9:2379 30459758 10.3389/fimmu.2018.02379 PMC6232773

[44] Taylor SL, Frias-Staheli N, García-Sastre A, Schmaljohn CS (2009) Hantaan virus nucleocapsid protein binds to importin alpha proteins and inhibits tumor necrosis factor alpha-induced activation of nuclear factor κB. J Virol 83:1271–1279 19019947 10.1128/JVI.00986-08 PMC2620888

[45] Cai M, Liao Z, Zou X, Xu Z, Wang Y, Li T, Li Y, Ou X, Deng Y, Guo Y, Peng T, Li M (2020) Herpes simplex virus 1 UL2 inhibits the TNF-α-mediated NF-κB activity by interacting with p65/p50. Front Immunol 11:549 32477319 10.3389/fimmu.2020.00549 PMC7237644

[46] Holloway G, Truong TT, Coulson BS (2009) Rotavirus antagonizes cellular antiviral responses by inhibiting the nuclear accumulation of STAT1, STAT2, and NF-κB. J Virol 83:4942–4951 19244315 10.1128/JVI.01450-08 PMC2682104

[47] Graff JW, Ettayebi K, Hardy ME (2009) Rotavirus NSP1 inhibits NFκB activation by inducing proteasome-dependent degradation of beta-TrCP: a novel mechanism of IFN antagonism. PLoS Pathog 5:e1000280 19180189 10.1371/journal.ppat.1000280 PMC2627925

[48] Zell R, Delwart E, Gorbalenya AE, Hovi T, King AMQ, Knowles NJ, Lindberg AM, Pallansch MA, Palmenberg AC, Reuter G, Simmonds P, Skern T, Stanway G, Yamashita T, Ictv Report C (2017) ICTV virus taxonomy profile: picornaviridae. J Gen Virol 98:2421–2422 28884666 10.1099/jgv.0.000911 PMC5725991

[49] Vreugdenhil GR, Wijnands PG, Netea MG, van der Meer JW, Melchers WJ, Galama JM (2000) Enterovirus-induced production of pro-inflammatory and T-helper cytokines by human leukocytes. Cytokine 12:1793–1796 11097750 10.1006/cyto.2000.0786

[50] Tung WH, Hsieh HL, Yang CM (2010) Enterovirus 71 induces COX-2 expression via MAPKs, NF-κB, and AP-1 in SK-N-SH cell: Role of PGE_2_ in viral replication. Cell Signal 22:234–246 19800403 10.1016/j.cellsig.2009.09.018

[51] Tung WH, Lee IT, Hsieh HL, Yang CM (2010) EV71 induces COX-2 expression via c-Src/PDGFR/PI3K/Akt/p42/p44 MAPK/AP-1 and NF-κB in rat brain astrocytes. J Cell Physiol 224:376–386 20333648 10.1002/jcp.22133

[52] Saura M, Lizarbe TR, Rama-Pacheco C, Lowenstein CJ, Zaragoza C (2007) Inhibitor of NF κB alpha is a host sensor of coxsackievirus infection. Cell Cycle 6:503–506 17351338 10.4161/cc.6.5.3918

[53] Shankar K, Sorin MN, Sharma H, Skoglund O, Dahmane S, Ter Beek J, Tesfalidet S, Nenzén L, Carlson LA (2024) In vitro reconstitution reveals membrane clustering and RNA recruitment by the enteroviral AAA+ ATPase 2C. PLoS Pathog 20:e1012388 39102425 10.1371/journal.ppat.1012388 PMC11326647

[54] Fang Y, Wang C, Wang C, Yang R, Bai P, Zhang XY, Kong J, Yin L, Qiu Y, Zhou X (2021) Antiviral peptides targeting the helicase activity of enterovirus nonstructural protein 2C. J Virol 95:e02324-e2420 33789997 10.1128/JVI.02324-20 PMC8315976

[55] Ji L, Yang E, He S, Jin Y, Chen D (2021) Enterovirus 2C protein suppresses IKKα phosphorylation by recruiting IKKβ and IKKα into viral inclusion bodies. Viral Immunol 34:218–226 33226912 10.1089/vim.2020.0173

[56] Alharbi KS, Fuloria NK, Fuloria S, Rahman SB, Al-Malki WH, Javed Shaikh MA, Thangavelu L, Singh SK, Rama Raju Allam VS, Jha NK, Chellappan DK, Dua K, Gupta G (2021) Nuclear factor-κB and its role in inflammatory lung disease. Chem Biol Interact 345:109568 34181887 10.1016/j.cbi.2021.109568

[57] Ouyang G, Liao Q, Zhang D, Rong F, Cai X, Fan S, Zhu J, Wang J, Liu X, Liu X, Xiao W (2020) Zebrafish NF-κB/p65 is required for antiviral responses. J Immunol 204:3019–3029 32321758 10.4049/jimmunol.1900309

[58] Guan H, Jiao J, Ricciardi RP (2008) Tumorigenic adenovirus type 12 E1A inhibits phosphorylation of NF-κB by PKAc, causing loss of DNA binding and transactivation. J Virol 82:40–48 17959673 10.1128/JVI.01579-07 PMC2224381

[59] Jobe F, Simpson J, Hawes P, Guzman E, Bailey D (2020) Respiratory syncytial virus sequesters NF-κB subunit p65 to cytoplasmic inclusion bodies to inhibit innate immune signaling. J Virol 94:e01380–20 32878896 10.1128/JVI.01380-20 PMC7592213

